# Calcium imaging in the ant *Camponotus fellah *reveals a conserved odour-similarity space in insects and mammals

**DOI:** 10.1186/1471-2202-11-28

**Published:** 2010-02-26

**Authors:** Fabienne Dupuy, Roxana Josens, Martin Giurfa, Jean-Christophe Sandoz

**Affiliations:** 1 Université de Toulouse; UPS; Research Centre for Animal Cognition (UMR 5169), 118 route de Narbonne, F-31062 Toulouse Cedex 9, France; 2 CNRS; Research Centre for Animal Cognition (UMR 5169), 118 route de Narbonne, F-31062 Toulouse Cedex 9, France; 3 Grupo de Estudio de Insectos Sociales, Departamento de Biodiversidad y Biologia Experimental, Facultad de Ciencias Exactas y Naturales, Universidad de Buenos Aires, Pabellon II, Ciudad Universitaria (C1428 EHA), Buenos Aires, Argentina; 4 CNRS; Evolution, Genome and Speciation (UPR 9034), 1 avenue de la Terrasse, 91198 Gif-sur-Yvette cedex, France

## Abstract

**Background:**

Olfactory systems create representations of the chemical world in the animal brain. Recordings of odour-evoked activity in the primary olfactory centres of vertebrates and insects have suggested similar rules for odour processing, in particular through spatial organization of chemical information in their functional units, the glomeruli. Similarity between odour representations can be extracted from across-glomerulus patterns in a wide range of species, from insects to vertebrates, but comparison of odour similarity in such diverse taxa has not been addressed. In the present study, we asked how 11 aliphatic odorants previously tested in honeybees and rats are represented in the antennal lobe of the ant *Camponotus fellah*, a social insect that relies on olfaction for food search and social communication.

**Results:**

Using calcium imaging of specifically-stained second-order neurons, we show that these odours induce specific activity patterns in the ant antennal lobe. Using multidimensional analysis, we show that clustering of odours is similar in ants, bees and rats. Moreover, odour similarity is highly correlated in all three species.

**Conclusion:**

This suggests the existence of similar coding rules in the neural olfactory spaces of species among which evolutionary divergence happened hundreds of million years ago.

## Background

A major aim of neuroscience is to understand how physical stimuli are represented in the animal or human brain, and to attempt to describe the main dimensions that define the perceptual spaces of these organisms [1]. As olfaction represents a key sensory modality in most animal species, numerous studies in the past have endeavoured to unravel the anatomical and functional features of olfactory centres, from the repartition of olfactory receptors at the periphery, their projection within the glomeruli of primary odour centers (olfactory bulb in vertebrates, antennal lobe in insects) and further projection to higher brain centers (olfactory cortex in vertebrates, mushroom bodies in insects) [2-5]. Neurophysiological studies, using both electrophysiological [6-11] and optophysiological techniques [12-18] have studied how different odour molecules differentially activate subsets of neurons or glomeruli. These studies have emphasized the remarkable similarities both in the general organization and in the odour coding properties of the olfactory systems of animals as remote in evolutionary terms as higher vertebrates and insects [19,20]. A general finding of these studies was that odours give rise to a combinatorial pattern of activity that can be measured across neurons of the same structure or across glomeruli in the antennal lobe (AL) or olfactory bulb. Moreover, odours sharing a chemical functional group or showing similar length of the carbon chain give rise to across-fibre patterns that are similar [21-23]. Therefore, similarity in the chemical world finds a representation in the neural activity of olfactory brain centres [24].

In the honeybee *Apis mellifera*, using an appetitive conditioning approach, we recently described the pair-wise perceptual similarity among 16 aliphatic odours from 4 different functional groups (primary and secondary alcohols, aldehydes and ketones) and 4 different chain lengths (from C6 to C9; [25]). Using multidimensional analyses, we could show that the main dimensions that defined bees' behaviour were indeed chemical dimensions like chain length and functional group of odour molecules. This study also showed that odours that induce similar calcium activity patterns in the bee AL are indeed treated by bees as similar in their behaviour. The logical conclusion from this work would be that chemical dimensions could represent essential dimensions encoding the representations of general odours (i.e. not including pheromones) in the brain and that odour similarity relationships should be relatively conserved in different species across the evolutionary scale. Now a number of species allow measuring neural olfactory similarity between odours, like rats and mice [15,26]), turtles [27], salamander [28], xenopus ([28-30], fishes [16,31], locusts [32], honeybees [12,33], ants [34], moths [35-37], drosophila [11,38,39], etc.

To start a comparative measure of odour similarity in different organisms, we have carried out optical imaging recordings in the olfactory system of a novel species of Hymenoptera, the ant *Camponotus fellah*. For such comparative studies, ants are an interesting model as they constitute a varied group with a great diversity of life histories, ecological interactions and novel evolutionary adaptations [40]. Olfactory cues are important in most aspects of their life, such as foraging, communication, larval grooming, nest defence and localization, social control and nestmate recognition. Moreover, the anatomical structures of the ant brain, in particular their olfactory circuits, are being described in great details [41-45] and they are amenable to electro- and optophysiological recordings in the brain [34,41,46]. Lastly, ants (in particular *Camponotus fellah*) can be individually trained to associate odours with gustatory reinforcers in a Y-maze under controlled conditions, allowing access to the study of the neural bases of olfactory learning and memory [47].

Adapting to these ants a method developed in the bee for specifically staining second-order AL neurons [33], we have measured the similarity among 11 aliphatic odours including alcohols, aldehydes and ketones, which were previously used for recording odour-evoked activity in the honeybee brain [48]. We show that these odours evoke activity in particular glomerular clusters of the ant AL and that these regions of the ant olfactory system classify odorants in a similar way as in the honeybee. Consequently, we show that odour similarity relationships are conserved among ants and bees. Moreover, using published data from radioactive 2-deoxyglucose (2DG) stainings on the olfactory bulb of rats [22], we show that odour similarity in ants is highly correlated to odour similarity in rats. Our result suggests that the main dimensions of ant, bee and rat olfactory spaces may be conserved, although evolutionary divergence among these species happened in the range of hundreds of million years ago. Therefore, although based on very different sensory receptors at the periphery, the olfactory systems of these animals would give rise, thanks to combinatorial coding, to similar perceptual relationships among odorants.

## Methods

### Preparation and staining

Worker ants of medium size (~7 mm) were taken from one of two colonies and were immobilized by cooling on an ice bed. They were mounted into Plexiglas chambers and their heads were immobilized with low-temperature melting wax (Deiberit 502, Böhme & Schöps Dental GmbH, Goslar, Germany). The antennae were then gently oriented to the front of the chamber and their base was fixed with two-component epoxy glue (Araldite) providing a seal between the flagella and the rest of the head. Then a small pool was created on top of the head by fixing thin plastic walls on the sides of the chamber (see [49]). A window was opened in the head cuticle, glands and tracheas covering the brain were removed. Throughout the preparation, the brain was regularly washed with saline solution (in mM: NaCl, 130; KCl, 6; MgCl_2_, 4; CaCl_2_, 5; sucrose, 160; glucose, 25; HEPES, 10; pH 6.7, 500 mOsmol; all chemicals from Sigma-Aldrich, Lyon, France). For calcium imaging experiments, we aimed to specifically stain the projection neurons that convey odor information from the AL to the mushroom bodies and the lateral horn. For that, we placed highly-concentrated chips of Fura-2 dextran (10000 MW, in bovine serum albumine - 2% in distilled water) on their axonal path [45], between the α-lobe and the border of the optic lobes, as was done in the honeybee [33]. We first used a sharp glass microelectrode (~30 μm tip diameter) to perforate the thick neurolemma at the chosen location. Then a second microelectrode was used to place the Fura-2 chips within the brain. After placing the dye, the brain was thoroughly washed with saline to remove extra-cellular dye. The piece of head cuticle was then replaced onto the opening and the ant was left in a dark place for three hours.

### Optical recordings of odor-evoked activity

*In vivo *calcium imaging recordings were carried out using a TILL photonics imaging system (Martinsried, Germany). Ants were placed under an epifluorescent microscope (Olympus BX-51WI) with a 10× (NA 0.3) or a 20× (NA 0.5) water immersion objective (UMPlanFL Olympus). Images were taken using a 640 × 480 pixel 12-bit monochrome CCD-camera (T.I.L.L. Imago) cooled to -12°C. Filters used were a 405 nm dichroic filter and a 440 nm emission filter. The preparation was alternately excited with 340 nm and 380 nm monochromatic light using T.I.L.L Polychrom IV. Each recording consisted of 100 double frames, at 5 double frames per sec. We used 4×4 (20× objective) or 2×2 (10× objective) binning on chip, so that pixel size in our recordings always corresponded to ~2 × 2 μm. Integration time was 4-40 ms and 10-128 ms respectively for 340 nm and 380 nm excitation. Odour stimuli were applied for 1 sec and started just before the 15^th ^double frame. Under the microscope, a constant air-stream was directed to the ant's antennae (2 cm distance). During odour stimulation, a secondary airflow was diverted from the main airflow and passed through an interchangeable glass pipette containing 4 μl of the odour on a 1 cm^2 ^filter paper. Over all animals, a range of eleven aliphatic odours was used: 1-*hexanol, 1-heptanol, 1-octanol, 1-nonanol, 2-hexanol, 2-octanol, 2-heptanone, 2-nonanone, hexanal, heptanal, octanal *(Sigma-Aldrich). Because in early experiments, a more limited range of odours was used, not all odours were tested in all animals. As control stimulus, a pipette containing a clean piece of filter paper was used. An experimental run consisted in 3 fully-randomised series of all stimuli with ~1 min intervals. A total of 79 ants were subjected to optical imaging experiments, out of which only 7 showed good calcium responses to odorants and allowed recording 3 complete odour stimulation runs. Compared to honeybees, we experienced more difficulty in ants for adequate staining of projection neurons and for the survival of animals through the experiment.

### Measures of glomerular layout

**Figure 1:**
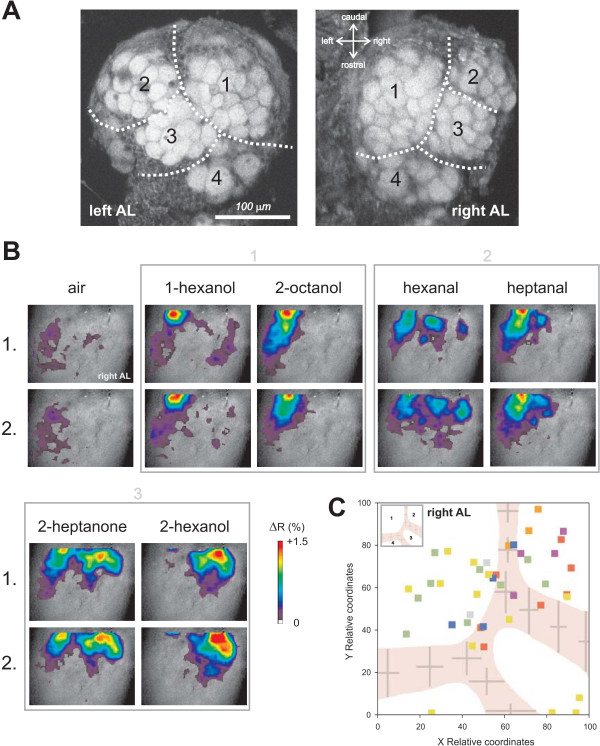
**Optical imaging of odour-evoked activity in the projection neurons of the ant *Camponotus fellah***. A) Z-projection of a confocal stack of the left and right antennal lobes of a worker ant showing the anatomical features accessible to optical imaging. The frontal side of the antennal lobe presents on average 46 glomeruli arranged according to 4 clusters. Cluster arrangement is symmetrical between brain hemispheres. B) Example of calcium signals from the projection neurons in the antennal lobe of a worker ant. Odour-evoked activity is superimposed on a wide-field image of the lobe, using a false-colour code, ranging from just above baseline (dark violet, +0.1% ΔR) up to maximal activation (red, +1.5% ΔR). Two stimulations with each odour and the air control show the reproducibility of the calcium signals. Squares with numbers 1 to 3 relate to different classes after the cluster analysis presented in figure 3A. C) Standard right antennal lobe of *C. fellah *workers in a relative coordinate system, showing the average borders of the different glomerular clusters (anatomical preparations, n = 16 lobes). The inset indicates the numbers given to each cluster (see A). Colour squares correspond to the active spots identified in 7 ants which showed reproducible calcium signals. Most active spots were recorded in the two caudal clusters 1 and 2.

During optical imaging, the glomerular structure of the ALs was hardly visible, so we performed additional stainings. The brains were thus treated according to a protocol that proved efficient in the honeybee: a mixture 125:1 (vol/vol) of a protease solution (from *Bacillus licheniformis *in propylene glycol, Sigma Aldrich), for digesting the brain sheath, and of the dye RH795 (dissolved in absolute ethanol), for staining cell membranes (Molecular Probes), was applied for 1 h. The brain was rinsed and fluorescent photographs were taken at 50-60 different focal planes under 530 nm excitation (filter set: 570 nm dichroic mirror and LP 590 nm emission filter). These images allowed seeing a few glomeruli and recognizing main landmarks on the AL, but failed to provide a precise glomerular layout. We thus performed a different type of staining on ants that were not subjected to imaging. As above, a protease was applied for 1 h. Then the brain was carefully rinsed with saline, and a filtered 4% neutral red solution in distilled water was applied for 1 h. The images, which were taken as above, clearly revealed the AL structure with its different glomerular clusters and allowed us to count and measure the glomeruli. We also used these anatomical data to construct a standard AL model, in which a few key anatomical landmarks (borders between the different glomerular clusters) were placed in a relative coordinate system. The × and Y coordinates of these landmarks measured in different individuals were averaged for constructing the standard AL model shown in Figure 1C. Thereafter, activity foci from imaging recordings could be placed in the same coordinate system to identify the most active areas of the ant AL. Note that the standard AL was not used for calculations of similarity between odor response patterns, or for comparisons among species. For additional anatomical reference, a few preparations were brought to a laser-scanning confocal microscope (Leica TCS SP2, 543 nm HeNe laser) and about 40 optical sections at the frontal surface of the AL were acquired with ~2 μm intervals. Z-projections (see Figure 1A) were made using the Image-J software (NIH, USA).

### Data processing and analysis

#### Raw data processing

Calcium-imaging data were analyzed using custom-made software written in IDL (Research Systems Inc., Colorado, USA). Each recording to an odour stimulus corresponded to a 4-dimensional array with the excitation wavelength (340 nm or 380 nm), two spatial dimensions (x, y pixels of the area of interest) and the temporal dimension (100 frames). Three steps were carried out to calculate the signals: First, to reduce photon (shot) noise, the raw data were filtered in the two spatial and in the temporal dimensions using a median filter with a size of 5 pixels. This step was applied separately for the 340 nm and the 380 nm data. Second, to correct for bleaching and possible irregularities of lamp illumination in the temporal dimension, a subtraction was made at each pixel of each frame, of the median value of all the pixels of that frame. Such a correction stabilizes the baseline of the recordings, without removing pertinent signals. This step was applied separately for the 340 nm and the 380 nm data. Third, for each pixel, the ratio R of the 340 nm and the 380 nm data was made and then ΔR was calculated at each frame, as ΔR = R_n _- R_0_, in which R_n _is the ratio data at frame n and R_0 _is a reference frame before the stimulus, here the average of frames 5 to 14. Thus, all ratios were close to 0 shortly before the odour stimulus.

#### Selection of activity spots

Because we had only limited anatomical information in ants subjected to calcium imaging, we developed a procedure for selecting the most relevant activity spots in each AL. We first made colour-coded activity maps for each odour in each animal, which allowed seeing neural activity spots. Activity spots were first selected visually, and activity was measured on a square of 9 × 9 pixels (18 μm × 18 μm), a surface that would be well within an individual glomerulus (see results). We then computed within each individual a measure of noise, as the standard deviation of the signal before the stimulus (from frame 4 to 11) averaged over all selected spots and all measurements in this animal [33]. Activity spots were only selected if the amplitude of excitation (or inhibition) was above (respectively below) the noise threshold. A spot needed to show significant activity to at least one odour to be selected at this stage. This activity also had to be reproducible over the three presentations of the odour. Because we used a ratiometric dye, an excitatory signal (an intracellular calcium increase) should induce a fluorescence increase when exciting at 340 nm and a decrease at 380 nm (the contrary for an inhibitory signal). We thus further inspected all selected signal curves, and only kept activity spots that showed the appropriate inversion of 340 and 380 nm signals upon odour delivery. This conservative procedure ensured that only biologically-relevant signals were taken into account in the analysis. For ensuring that the selection of activity spots was unbiased, the person carrying out this selection was blind with respect to the tested odours and to the statistical analyses that would be performed.

#### Measures of odour similarity and comparison among taxa

**Figure 2:**
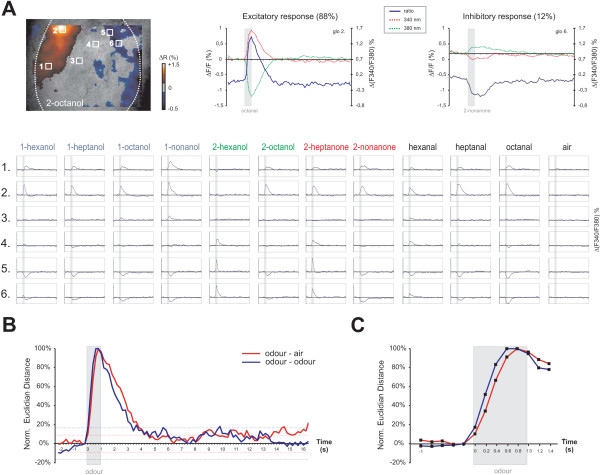
**Time courses of calcium signals in the projection neurons of ants**. A) Time courses obtained in 6 glomeruli as in Figure 1B, after stimulation with 11 aliphatic odours and the air control. Odour names are indicated in colour according to their functional group (aldehydes, black; ketones, red; primary and secondary alcohols, blue and green respectively). Odour delivery (1 sec) is indicated as a grey bar. Both excitatory (calcium increase) and inhibitory (calcium decrease) signals were observed, as well as a few temporally complex signals. On the upper left, an activity map (2-octanol) shows both excitatory responses (in shades of red) and inhibitory responses (in shades of blue) on the same lobe. On the upper right, two examples of typical time courses for excitatory and inhibitory signals are given. In each case, an inverse evolution of fluorescence recorded with 340 nm and 380 nm excitations is observed. B) Time course of a measure of odour separability in all recorded ants. The instantaneous Euclidian distance between each odour glomerular pattern and the air control (red curve) gives an indication of how fast the ant olfactory system can best separate an odour from an odourless background (~800 ms). The instantaneous Euclidian distance between the activity patterns obtained for any two odours (blue curve) gives an indication of how fast the ant olfactory system reaches an optimum in its separation power among odours (~600 ms). Both distances are normalized to 1 at their maximum and to 0 just before odour onset. For this reason, they can be under 0 just before the stimulus. C) Same data as B, showing in greater details the evolution of both measures during the stimulus.

One aim of this study was to measure similarity between odorants based on the signals obtained in the ant AL. To do that, we used the Euclidian distance between odour representations in a n-dimensional space in which the activity of each spot represents one dimension [12,49,50]. The higher the distance, the less similar odour representations are. We included in this analysis only ants that showed at least 4 activity spots (n = 5 ants). First, we asked at which point in time the discriminability among odours reaches a maximum. During a recording, activity for each odour follows an individual trajectory in the space of neural activation. Instantaneous discriminability between stimuli can thus be measured as the distance between odour representations at each point in time. We thus computed within each animal, and at each double-frame, Euclidian distances between odour representations for all odour pairs. The time courses of two types of distances were thus obtained: (i) the mean distance between each odour and the air control; (ii) the mean distance between any two odours tested. These distances were first averaged at each double-frame within each animal, and then over different animals. To show the time courses in the same figure (Figure 2C, D), the curves were scaled to 0% just before odour delivery (frame 14) and to 100% at their own maximum. As frame 18 (~600 ms after odour delivery) proved to be the optimum for measuring inter-odour distances, all further calculations were carried out on response amplitudes measured between just before the stimulus (average of frames 12, 13 and 14) and around frame 18 (average of frames 17, 18 and 19).

**Figure 3:**
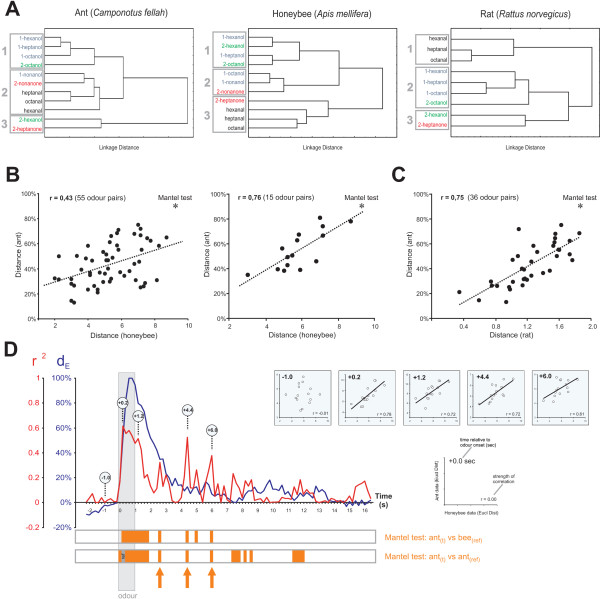
**Comparison of odour similarity in ants, bees and rats**. A) Dendrogram (Ward's classification) showing similarity relationships among the 11 aliphatic odours in ants, honeybees *Apis mellifera *(data from [48]) and rats *Rattus norvegicus *(9 odours, data from [22]). Three main clusters are found in each species, with one cluster containing mainly alcohols (cluster ant #1, bee #1 and rat #2), and another containing the three aldehydes (cluster ant #2, bee #3, rat #1). B) Inter-odour Euclidian distances in ants as a function of the same measure in honeybees, with either maximized number of odour pairs (Left, 55 odour pairs, 3-5 ants, r = 0.43, Mantel test, p = 0.036) or maximized number of ants (Right, 15 odour pairs, 5 ants, r = 0.76, Mantel test, p = 0.018). C) Inter-odour distances in ants as a function of the same measure in rats (36 odour pairs, 3-5 ants, r = 0.75, Mantel test, p < 0.001). D) Running correlation between instantaneous Euclidian distances in ants (maximizing ant number, as in B, right panel) and fixed-point distances in honeybees [48]. The quality of the correlation (r^2 ^in red) is plotted along with the inter-odour distance (in blue, taken from Figure 2B) showing the separation power of the ant olfactory system throughout a recording. Maximum correlation is obtained very shortly after odour application (~200 ms), and remains high throughout odour presentation. After odour offset, correlation decreases but oscillations are observed, with high correlation epochs as late as 5 sec after odour application, when activity and the separation powerof the ant system are low. Orange bars below the graph indicate significance in running Mantel tests 1) between ant data at each 200 ms time bin and the fixed-point bee data or 2) between ant data at each 200 ms bin and fixed-point ant data 200 ms after odour onset (frame termed 'ref' in the figure). Significant correlation rebounds (see text) are marked with orange arrows. Circles correspond to plots between ant and bee data on the upper right, showing instantaneous Euclidian distances in the ant as a function of distances in the bee.

Within each ant, a matrix of inter-odour distances was thus calculated. We set to 100% the highest distance of each animal, and scaled all other distances accordingly. To ask whether odour-similarity relationships are the same in different animal species, we subjected our dataset to correlation analyses with previous data on honeybees [48] and on rats [22]. Distances between odour representations in the bee data were calculated as in [25]. AL activation maps (as presented on http://neuro.uni-konstanz.de/) were transcribed into activation levels for each glomerulus from 0 to 3 according to the following signal scale: activity below 40%: 0; 40-60% activity: 1; 60-80% activity: 2; >80% activity: 3. Since not all ants could be tested with all odours, we performed two different analyses. In the first one, we maximized the number of odour pairs evaluated, so we used all 11 aliphatic odours, and the distances obtained from 3 to 5 ants were averaged for each of the 55 odour pairs. In a second analysis, we maximized the number of ants, so we used only 6 aliphatic odours that were tested in all 5 ants, and evaluated respectively 15 odour pairs. Both analyses gave essentially the same result. In the rat, Johnson *et al*. [22] mapped 2DG responses to a wide range of aliphatic odorants, including 9 odours used in the present study, onto 13 identified lateral modules (groups of glomeruli) and their 13 homologous medial modules. We transcribed activity intensity depicted as circles in Figure 2 of Johnson *et al*. [22] to percentages of activity in each module (from 0% to 100%, in 10% steps) to each of the 9 odours (*hexanal, heptanal, octanal, 1-hexanol, 1-heptanol, 1-octanol, 2-hexanol, 2-octanol, 2-heptanone)*. The matrix of Euclidian distances between these odour representations was used for a correlation analysis as with bee data, maximizing the number of odour pairs (3-5 ants, 36 odour pairs). In the present paper, data from the lateral modules of the olfactory bulb are shown (Figure 3C), but data from the medial modules give the same result (data not shown). To test for the significance of the correlation coefficients observed between distance matrices in two species, the Mantel test, a dedicated random permutation test, was used [51]. This test randomly permutes the distance values within one of the two matrices and then computes the correlation coefficient with the permuted data. By doing so many times (here we used 10000 permutations), the alpha value for the significance of the correlation coefficient is directly estimated. Ant, bee and rat distance matrices were also used in cluster analyses to represent groups of similar odours, using Ward's classification method.

## Results

### Anatomy of the frontal surface of the antennal lobe of *C. fellah*

The front part of the AL of *C. fellah*, which is accessible to optical imaging, contains mostly four different glomerular clusters, defined by thin but clear clefts between groups of glomeruli (Figure 1A). We analysed 16 ALs from 8 different ants, and found an average of 46.0 ± 2.6 (mean ± SEM) visible glomeruli on the frontal surface of each lobe. Left and right ALs were clearly symmetrical, with the same overall number of glomeruli (45.7 ± 3.2 and 46.2 ± 4.4 respectively, t test, t = 0.41, NS) and the same structure in separate glomerular clusters (Figure 1A). For this reason, and because imaging was performed mostly on right ALs, all subsequent analyses were carried out as for right ALs, left ALs being flipped horizontally. Glomerulus size was rather constant, and width for instance varied maximally with a factor 2, as the smallest glomerulus had dimensions of 20.2 ± 1.2 μm × 17.6 ± 0.7 μm and the largest glomerulus dimensions of 38.0 ± 1.7 μm × 39.1 ± 1.8 μm (n = 16 lobes). The four clusters were easily recognizable and arranged similarly in different individuals. Cluster 1 was the largest, was placed on the medio-caudal side and contained on average 18.1 ± 0.8 glomeruli. Clusters 2, 3 and 4 followed a diagonal axis on the lateral side of the AL and contained 10.4 ± 0.9, 8.4 ± 0.7 and 5.6 ± 1.0 glomeruli respectively. Based on this anatomical data, we constructed a standard AL, in which the border between the different clusters is represented as averages ± SD (Figure 1C, inset) in a relative coordinate system based on each lobe's width (X) and height (Y) in the picture. The standard AL will be used for placing activity spots from the imaging data.

### Odour responses from projection neurons

Seven ants (out of 79 tested animals) showed good calcium responses to odorants, which allowed recording of 3 complete odour stimulations runs. In such individuals, odour stimulation led to specific activity patterns (Figure 1B), which comprised mostly excitatory responses (intracellular calcium increase). Odour responses were reproducible as the same pattern appeared when the same odour was presented again (Figure 1B). Different odours induced signals in a different combination of activity spots. The size of active spots, as measured from the activity maps was on average 14 pixels (~28 μm), which corresponds roughly to the size of individual glomeruli, as observed above. When recording the relative position of activity spots of the 7 ants and placing them on our standard *C. fellah *AL, it appears clearly that signals were mostly obtained from two regions on the caudal side, corresponding to Clusters 1 and 2. Except in one individual, in which activity was found in the rostral part of the lobe (yellow individual in Figure 1C), Clusters 3 and 4 were mostly silent.

**As indicated above**, most recorded responses were of the excitatory type, as in 88% of the cases, ΔR increased upon odour delivery (Figure 2A). In 12% of the cases, however, response was inhibitory, as ΔR decreased clearly upon odour delivery. In each case, the ΔR responses reflected an inverse evolution of fluorescence recorded at 340 nm and 380 nm (Figure 2A, upper right), indicating that they correspond to calcium concentration changes within the recorded neurons. Excitatory responses showed different types of time courses, ΔR reaching a maximum as quickly as 200-400 ms after odour onset (glomerulus 5, 2-heptanone), while in some other cases, a maximum was reached several hundred ms after the end of the stimulus (glomerulus 1, 1-nonanol). Most responses had a phasic-tonic shape, with a slow return to baseline after stimulus offset (several seconds, glomerulus 2, octanal), and in some cases a prolonged tonic component (glomerulus 2, 1-nonanol). In a few cases, responses were clearly phasic, coming back to baseline very shortly (~1 sec) after stimulus offset (glomerulus 5, 2-hexanol, 2-heptanone). Inhibitory responses were usually slower, reaching a minimum as early as 600-800 ms after odour onset (glomerulus 5, hexanal) - but also as late as 1 s after odour offset (glomerulus 5, 1-octanol). In a few cases (<2%), the observed responses were temporally complex, showing first an excitatory phase and then an inhibitory phase (for instance, glomerulus 6, 1-hexanol) or the contrary (data not shown). Depending on the presented odour, a same glomerulus could present both excitatory and inhibitory responses, with even sometimes complex responses. For instance, in Figure 2A glomerulus 6 responded with an excitation to 2-hexanol, 2-heptanone and hexanal, while it responded with an inhibition to most other odours, and with a biphasic response to 1-hexanol.

The neural representation of an odor can be regarded as a vector in a multidimensional space, in which each dimension is represented by a particular glomerulus. Given the heterogeneous nature of the time courses of glomerular responses, we asked how this heterogeneity translates in terms of discrimination power of the ant olfactory system. We therefore computed two different measures of discriminability among stimuli (Euclidian distance) throughout a recording. First, we asked at which point in time the neural activity induced by odorants is the most salient, i.e. when the Euclidian distance between the representation of an odour and the air control is the highest. Second, we asked at which point in time different odours could be separated most efficiently, i.e. when the Euclidian distance between odours representations is the highest. When considering the average odour-air distance (Figure 2B-C), we found that it increased throughout odour presentation, from 10% just after odour onset, to 67% of its maximum ~400 ms after odour onset, reaching 100% after 800 ms, before decreasing slowly towards baseline several seconds after odour offset. When following the inter-odour distance, we found a similar time course, reaching 52% of its maximum ~200 ms after odour onset, and 100% after 600 ms. This distance also decreased slowly, returning to baseline several seconds after odour offset. These two measures indicate that the ant olfactory system can detect odours (odour-air distance) and separate them (inter-odour distance) within a few hundred milliseconds after odour onset, and that such odour separation can last for a few seconds after odour offset.

### Comparison of neural olfactory spaces in ants, bees and rats

Using multidimensional scaling techniques, we asked how the ant olfactory system classifies the 11 aliphatic odours. A cluster analysis based on Euclidian distances between odour representations (Ward's classification) grouped odours according to their chain length and/or functional group (Figure 3A). One group contained shorter-chain primary alcohols and 2-octanol. A second group contained all three aldehydes together with two 9 carbon odours: 1-nonanol and 2-nonanone. A third, isolated group contained 2-hexanol and 2-heptanone. We compared this classification with those obtained in another hymenopteran insect, the honeybee *Apis mellifera *(data from Sachse *et al*. [48]). In the honeybee, we found also three main clusters, two of which were similar to their counterparts in *C. fellah*: a first group contained (mostly short chain) primary and secondary alcohols, with three common odours with its ant counterpart (1-hexanol, 1-heptanol and 2-octanol). A second group contained two long-chain alcohols and 2-nonanone (two common odours with cluster 2 in the ant). A third group contained all three aldehydes and 2-heptanone. All three aldehydes were together in cluster 2 in the ant. To assess similarity between the two datasets, we represented Euclidian distances between odour representations in the ant as a function of the same measure in the bee (Figure 3B). When using all possible odour pairs between 11 aliphatic odours (Figure 3B, 55 odour pairs, between 3 and 5 ants per combination), we found a significant correlation between ant and honeybee similarity measures (r = 0.43, Mantel test, p = 0.036, n = 10000 repetitions), but with a rather broad distribution along the main axis. When maximizing the number of measured ants, thereby reducing the number of odour pairs (Figure 3B, 15 odour pairs, 5 ants per combination) we also found a significant correlation (r = 0.76, Mantel test, p = 0.018, n = 10000 repetitions) but with a much narrower distribution around the main axis. This analysis thus shows that an odour that is similar in the ant AL is also similar in the bee AL, and vice versa.

We then asked how the ant similarity matrix relates to a similar measure in rats (data from Johnson *et al*. [22]). Using a similar cluster analysis on the 9 odours in common with our study, we found that mainly three clusters appeared (Figure 3A): a first cluster contained all three aldehydes. A second cluster grouped primary alcohols and 2-octanol. A third cluster contained 2-hexanol and 2-heptanone. This clustering was very similar to that found in ants, which grouped the same odours in three clusters (except for two additional odorants that were missing in the rat data). To assess similarity between the two datasets, we represented Euclidian distances in the ant as a function of those in the rat (Figure 3C). We found a very highly significant correlation between ant and rat similarity measures (Figure 3C, 36 odour pairs, 3-5 ants, r = 0.75, Mantel test, p < 0.001, n = 10000 repetitions). The same analysis could not be carried out maximizing the number of ants (as Figure 2B, right panel), because only four odours were in common in the two datasets. This analysis shows that an odour that is similar in the ant AL is also similar in the rat olfactory bulb, and vice versa.

All previous comparisons of odour similarity matrices between animal models concentrated on one moment of odour responses in the ant, namely when the separation power of the olfactory system was maximum (400-800 ms after odour onset). To check the validity of the obtained correlations, we wanted to evaluate how this correlation evolves throughout a recording. We thus performed a running correlation of the ant data at each point during a recording with the fixed-point bee data (as above, distances based on the activity pattern during odour presentation, [48]). Before odour onset, the correlation between data sets was very low due to the lack of odour-coding information in the ant data. Within 200 ms after odour onset, the correlation strongly increased to a maximum of r^2 ^= 0.62 (r = 0.78) and remained stable throughout odour delivery. Mantel tests confirmed that this correlation was significant from 200 ms after odour onset, until 1 sec after odour offset (orange bars, Figure 3D bottom). After odour offset, correlation decreased showing a number of rebound epochs, during which a strong correlation between ant and bee data appeared again. At the four highest rebounds, between 1.6 and 5.0 sec after odour offset, the Mantel tests again indicated significance. Interestingly, these rebounds happened at a moment when most odour-induced activity (as measured by the average Euclidian distance between odour pairs, blue curve, Figure 3D) was again low. To confirm the validity of these correlations, we performed running Mantel tests to compare *within the ant dataset*, the Euclidian distance matrix between odours observed at each frame, with that observed at correlation maximum (200 ms after odour onset, termed 'ref' in Figure 3D, bottom). Logically, this analysis showed a high coherence of the ant inter-odour distance matrix throughout odour presentation and for 1 sec after odour offset (orange bars Figure 3D bottom). In addition, it showed a number of correlation rebounds at different moments after odour offset, from 1.6 sec until 10 sec afterwards. Three out of four of the correlation rebounds between ant and bee data (orange arrows in Figure 3D) corresponded to significant correlations within the ant dataset. This confirms that when neural activity in projection neurons is near baseline again, as late as 5 sec after odour offset, it still contains odour-specific information which correlates both with the odour code observed during odour presentation in ants and with the code found in honeybees. Such rebounds are reminiscent of a short-term sensory memory described in the bee by Galan *et al*. ([52]- see discussion).

## Discussion

We have imaged odour-evoked activity from second order olfactory neurons in the ant *Camponotus fellah*, and described glomerular activity for 11 aliphatic odours taken among alcohols, aldehydes and ketones. We show that the rules underlying odour similarity relationships in this ant species are similar to those found in another hymenopteran insect, the honeybee, but also in a mammal, the rat.

### Calcium signals in projection neurons

Calcium signals recorded upon odour delivery were mainly of two types. Most responses (88%) were excitatory, and showed a quick calcium increase at odour onset and slower return to baseline at odour offset. The time needed to reach a maximum varied across glomeruli and/or odours. This corresponds well to excitatory calcium responses obtained from projection neurons in bees ([33,53,54], Deisig *et al*. *subm*), drosophila [55,56], moths [57] and another ant species [41]. The second type of responses (12%) corresponded to negative responses, during which the fluorescence ratio decreased within projection neurons, usually with a slower dynamic than for excitatory responses. This result fits well with observations in bees [33,53], in which projection neurons receive inhibitory input from local interneurons. Recordings of individual PNs in the bee have shown that reduced firing during an odour stimulus (below the spontaneous firing frequency of the PN) is linked to such negative calcium responses [58].

### Measuring projection neuron subpopulations

Because we inserted the dye crystals laterally from the α lobe in direction of the optic lobes, as done previously in honeybees [33,53,59], we have potentially stained both populations of projection neurons that convey information from the AL to the mushroom bodies and the lateral horn, the lateral and the medial antenno-cerebralis tracts (l-ACT and m-ACT respectively). At the injection location, both tracts run in parallel but in opposite directions (bees: [60,61], ants: [41]). In *Camponotus *ants as well as in honeybees, l-ACT and m-ACT neurons are *uniglomerular *(each neuron takes information within only one glomerulus) and innervate two separate sub-populations of glomeruli. Almost all glomeruli convey their information to the mushroom bodies via either the l-ACT or the m-ACT, supporting the idea of a double parallel olfactory pathway in Hymenoptera [41,60-62]. So why did we find mostly signals on the caudal part of the lobe (Clusters 1 and 2, Figure 1C)? Several explanations may be given. First, inhomogeneous staining of the glomeruli could be responsible for this result. Since the distance to reach the AL from the injection location was much shorter for l-ACT neurons than for m-ACT neurons and migration time was relatively short (3 h), it could be that mostly l-ACT neurons (and their respective glomeruli) were stained. In our experiments, however, we did not observe any heterogeneity in average fluorescence intensity between caudal and rostral lobe regions. A second explanation would be related to the range of odours we have tested, and to a possible chemotopy of glomerular clusters, as found in vertebrates (e.g [22,23]): some glomerular clusters may be more sensitive to aliphatic alcohols, aldehydes and ketones, and other clusters more sensitive to aromatics, esters, acids, etc. This possibility is emphasized by the case of honeybees, in which the glomeruli of the frontal region, which are usually imaged, respond to some odour classes (among which alcohols, aldehydes and ketones), but not to some other classes, like alkanes or carboxylic acids [48]. However, other glomerular regions of the bee AL most probably do. The use of a wider range of odour types in future imaging experiments would help elucidating this question.

### Conserved odour similarity space: an emerging property of multiple channels

Electrophysiological and/or optophysiological measures of odour responses have been obtained in the AL of insects or the olfactory bulb of vertebrates (see introduction). We have chosen two datasets that provided many common odours with our study: calcium imaging recordings in the honeybee AL [48] and 2DG stainings in the rat olfactory bulb [22]. We found highly significant correlations between ant and bee data, but also between ant and rat data. In other terms, odours we found to be similar for the ant olfactory system, were also similar for bees' and for rats' olfactory systems. This in turn suggests that odour representation in the olfactory space of these animals - although based on systems with differing dimensions (numbers of glomeruli, etc), measured using different techniques (*in vivo *imaging, 2DG stainings), with different stimulus durations (1 sec in ants, 2 sec in bees, 45 min exposure in rats) - is similar. This emphasizes the robustness of the rules of odour coding in animals as diverse as insects and mammals. How should we interpret this finding? One evaluates the divergence between vertebrates and arthropods to have taken place about 550 to 830 million years ago [63,64]. Divergence between bees and wasps/ants is supposed to have been ~150 million years ago [65]. Although the architectures of the olfactory nervous systems of vertebrates and insects show fascinating similarities [19,20], especially in the glomerular modularity of AL and OB, recent data suggest that olfactory receptor (OR) proteins in insects and vertebrates are unrelated [66]. In other words, insect ORs constitute a family of G-coupled transmembrane receptors unknown in vertebrates. Therefore, we cannot attribute the highly significant correlation between ant and rat olfactory spaces to similarities in their OR repertoire. The same logic may even apply to the comparison between ants and bees, although confirmation will need thorough analysis of ant OR repertoire. Indeed, all insects are thought to possess ORs belonging to the same receptor family, but within the genomes of the few insect species that have been sequenced until now, OR sequences are highly variable, with very few orthologs found between species. For instance, the honeybee genome contains ~163 functional ORs with very divergent sequences, which are mostly unrelated to those expressed in the fruitfly *Drosophila melanogaster*, the moth *Heliothis virescens *or the mosquito *Anopheles gambiae *[67]. Similarly, *Anopheles *and *Drosophila *ORs show very few ortholog receptors, although they both belong to Diptera [68]. Consequently, the sensory equipment for detecting odorants at the periphery is rather different in the three species. We therefore believe that the high correlations found between odour similarity matrices in our work are emerging properties of the multiple coding channels of each system (the ORs) which each detect a different but overlapping range of odorant molecule features. Individual coding units are different, but at the multidimensional level, they give rise to representations that mirror the chemical characteristics of the molecules, here chain length and functional group. Recently, we have started a description of the honeybee olfactory perceptual space, evaluating their behavioural generalisation performance among 16 aliphatic odorants differing systematically in functional group (primary and secondary alcohols, aldehydes, ketones) and carbon chain lengths (from 6 to 9 carbons) [25]. In fact, odour similarity measured in the behaviour correlated well with odour similarity measured in the AL by calcium imaging [48]. Multidimensional analysis of the behavioural data clearly showed that three main factors were the basis for the olfactory perceptual space underlying bees' responses. The first factor represented chain length information, while the second and third factors segregated functional groups. Therefore, the main properties of the odour molecules were defining bees' perceptual space. In the present case, using cluster analysis to group odorants on the basis of glomerular (ant and bee) or modular (rat) information showed very clear similarities: in each species, one cluster grouped all aldehydes together, and another cluster grouped most primary and secondary alcohols (Figure 3A). The clear grouping of all aldehydes together, found in all species, is reminiscent of the very strong behavioural generalisation we have found among odorants of this class in bees [25]. All these observations suggest that across species, and albeit different peripheral OR equipment, molecules with different functional groups and chain lengths will give rise to organized and segregated neural representations. We thus expect to find similar correlations of ant, bee, and rat data with still other animal models.

The running correlation we have performed between the matrices of inter-odour distances in the ant at each frame, and the distance matrix obtained previously in the bee (Figure 3D) has confirmed the robustness of this result. First, the high correlation found between data sets first appears upon odour delivery (r = 0.78, 200 ms after odour onset). Second, throughout odour delivery, the correlation remains at the same high level, decreasing only after odour offset. It is important to notice that the correlation found between ant and bee data was not limited to the periods of highest separation power of the olfactory system, since it appeared as soon as odours were presented to the ant, i.e. when separation power was only at ~50% of its maximum. Moreover, it strongly decreased just after odour offset, although separation power was still high (more than 60%, see + 2 sec after odour onset, Figure 3D). This suggests that the potential separation power described by the inter-odour distance, which lasts several seconds, does not carry the same information throughout PN activity: during odour stimulation, PN activity shows an output formatted by AL network activity, which loses its coherence about 1 sec after odour offset, resulting in a decreased correlation between ant and bee data. An interesting finding is the appearance of rebound effects several seconds after odour offset (Figure 3D). Thus, although the coding power within the calcium signals is almost down to baseline (see inter-odour distance in Figure 3D), a clear correlation is found between bee and ant data at these stages. This effect is reminiscent of rebound effects found in recordings of projection neurons in honeybees [52], in which glomeruli that were jointly activated by a given odour stimulation, retained an increased probability of being spontaneously active at the same time, in the next minutes after odour application. Therefore, the olfactory system would keep through spontaneous activity a kind of short-term sensory memory of an odour previously presented [52,69]. Although, our observation was in the range of seconds after the odour, such a phenomenon could explain why a pattern of inter-odour similarities that is similar to that obtained during odour stimulation would appear at a time when overall activity is near baseline (see Figure 3D). Because of this, reverberations of bee-ant correlations would appear.

The present study has shown the important robustness of general odour representation in animals that, because they live in similar worlds - the air medium - came to organize odour information in the brain in a similar way. The correlations found in this study were not perfect, however, and future work will have to understand how much of the observed scatter is due to experimental noise (recording method, regions recorded from, stimulus duration, etc.) and which part relates to the adaptation of each animal species to its particular environment. Obvious departures from such general rules will be the case of pheromonal odours, which are mostly processed by separate olfactory subsystems. We hope the present work will stimulate future comparative studies of neural odour representation in animals.

## Authors' contributions

FD carried out the optical imaging recordings and performed initial analysis. RJ and MG participated in the design of the experiments and of the analyses and to the final version of the manuscript. JCS conceived and coordinated the study, analyzed the data and wrote the manuscript. All authors read and approved the final version of the manuscript.
